# Detection of Conjugation Related Type Four Secretion Machinery in *Aeromonas culicicola*

**DOI:** 10.1371/journal.pone.0000115

**Published:** 2006-12-27

**Authors:** Ashraf Yusuf Rangrez, Kannayakanahalli Maheshwarappa Dayananda, Santosh Atanur, Rajendra Joshi, Milind S. Patole, Yogesh S. Shouche

**Affiliations:** 1 Molecular Biology Unit, National Centre for Cell Science, Pune University Campus, Pune, Maharashtra, India; 2 Centre for Development and Advanced Computing, Pune University Campus, Pune, Maharashtra, India; Institute for Genomic Research, United States of America

## Abstract

**Background:**

*Aeromonas* sp. can now be considered relatively common enteropathogens due to the increase of diseases in humans. *Aeromonas culicicola* is a gram negative rod-shaped bacterium isolated for the first time from the mosquito mid-gut, but subsequently detected in other insects and waters also. Our previous study discovered that *A. culicicola* harbors three plasmids, which we designated as pAc3249A, pAc3249B and pAc3249C. We investigated and report here the existence and genetic organization of a Conjugal Type IV Secretion System (TFSS) in pAc3249A.

**Methodology/Principle Finding:**

The complete operon is 11,061 bp in length and has G+C content of 47.20% code for 12 ORFs. The gene order and orientation were similar to those found in other bacteria with some differences. We have designated this system as *AcTra* for *Aeromonas culicicola* transfer system. BLAST results of ORFs and phylogenetic analysis showed significant similarity towards the respective proteins of the IncI2 plasmid R721 of *E. coli*. Other bioinformatics studies have been performed to predict conserved motifs/domains, signal peptides, transmembrane helices, etc. of the ORFs.

**Conclusions/Significance:**

BLAST results of ORFs and phylogenetic analysis showed significant similarity towards the respective proteins of the IncI2 plasmid R721 of *E. coli*.

## Introduction

*Aeromonas* are Gram-negative rod shaped facultative anaerobic bacteria of the family Aeromonadaceae. They are widely distributed in variety of habitats ranging from fresh water to salt water and found in virtually all foods. The past decade has witnessed an explosion of scientific interest in members of the genus *Aeromonas* and this interest has gone beyond fish pathogenicity. They are now considered as emerging human pathogens suspected to cause gastroenteritis ranging from mild enteritis to cholera like diarrhea [1], [2]. *A. hydrophila, A. caviae and A. veronii* represents more than 85% of clinical isolates [3], [4].

Several secretion machineries present in Gram-negative bacteria mediate the transport and injection of toxic molecules into target cells. These secretion systems are classified into five types I to V depending on similarities, differences and substrate specificities. These machineries share a common requirement for proteins that utilize ATP as an energy source to drive transport of macromolecules. TFSSs have long been recognized as the systems ancestrally related to bacterial conjugation machines and responsible for the exchange of genetic material [5]. By facilitating conjugative transfer, type IV secretion machineries play crucial roles in the spread of antibiotic resistance genes among bacteria. The bacterial TFSS mediate the transport of macromolecules across the cell envelope of Gram-negative and Gram-positive bacteria [6], [7]. Several important human and plant pathogens have evolved type IV secretion machineries involved in delivering virulence factors (proteins or protein-DNA complexes) to host target cells. The TFSS of *Agrobacterium tumefaciens* is prototype and is proved to be involved in crown gall disease [8]. Other pathogens are *Bordetella pertussis*, the agent responsible for whooping cough in children; *Helicobacter pylori*, responsible for gastric ulcers and stomach cancer; *Brucella suis*, the causative agent of brucellois and *Legionella pneumoniae*, the causative agent of Legionnaires' disease were shown to adopt TFSS for virulence [9]–[13]. The phylogenetic and functional relationships evident between type IV and certain conjugal transfer systems has led to the suggestion that these groups form a type IV superfamily of proteins involved in both the conjugal transfer between bacteria and the transit of virulence factors between bacteria and their eukaryotic host [14]. There was only one report of conjugal transfer system in *Aeromonas* before our study and that was found in a plasmid pFBAOT6, originally isolated from a *strain* of *A. caviae* from hospital effluent [15].

In this study, we characterized the sequence of conjugal TFSS from the plasmid pAc3249A of *A. culicicola* MTCC 3249. This *strain* harbors three plasmids of different sizes which we designated pAc3249A, pAc3249B and pAc3249C. pAc3249A is circular and the largest, approximately 30 kb in size, found to code for this conjugal transfer system. pAc3249B and pAc3249C are circular, approximately 8.5 kb and 3 kb in size respectively.

## Materials and Methods

### Bacterial strains and plasmids

*Aeromonas* species were maintained on Luria Bertani (LB) medium at 30°C. *E. coli* strains were maintained on LB at 37°C. When required, media were supplemented with ampicillin (100 µg/ml), and tetracyclin (25 µg/ml).

### DNA Manipulations

Plasmids were isolated using a Qiagen midi-prep plasmid isolation kit (Qiagen), followed by gel extraction after electrophoresis of the entire plasmid isolation eluate. Restriction endonuclease digestions, ligation, agarose gel electrophoresis were carried out as described in Maniatis et al. [16].The plasmid pAc3249A was digested with *Bam*HI, *Sau*3AI, *Hind*III, *Alu*I (New England Biolabs) and ligated into pLitmus29 (New England Biolabs) prepared by respective restriction enzymes digestion and dephosphorylation with Shrimp Alkaline Phosphatase (Roche). Ligated product was transformed into JM 109 competent cells (Invitrogen).

### DNA Sequencing and Sequence Assembly

DNA sequencing was carried out on an Applied Biosystems 3730 DNA Analyzer with an ABI PRISM BigDye Terminator cycle sequencing kit (Apllied Biosystems). The clones were initially sequenced with M13 forward and reverse vector specific primers. The sequences were analyzed and assembled using in-house assembly pipeline, which is integration of phred, cross match and phrap. Gaps between contigs were filled by primer walking.

### Web Servers and Homology Predictions

Putative coding sequences (CDSs) were identified using glimmer [17]. Functional annotation was done by searching putative protein coding sequences against non-redundant protein database obtained from NCBI using BLASTP [18], against Pfam [19] using hmmer and ProDom [20] database. To identify the functional motifs, each CDS was searched against Prosite [21] database. Secondary structure, disulfide bridges, globularity, and non-standard secondary structure about each putative protein was obtained by using PredictProtein [22] software. The sequence homologs were obtained by PSI-BLAST. Multiple sequence alignment of homologous sequences was carried out using ClustalW [23]. Bootstrap analysis was carried out using SEQBOOT to generate 100 random combinations of the alignments in Phylip. Phylogenetic trees (cladogram) were constructed using parsimony method of Phylip and trees were visualized using TREEVIEW 16. TMHMM server version 2.0 (http://www.cbs.dtu.dk/services/TMHMM-2.0/) and DAS servers (http://www.sbc.su.se/~miklos/DAS/) were used to perform TMS prediction of all the genes, whereas signal peptide prediction was carried out using SignalP3 (http://www.cbs.dtu.dk/services/SignalP/) and LipoP1 (http://www.cbs.dtu.dk/services/LipoP/). Cello version 2.5 (http://cello.life.nctu.edu.tw/) and PSORT (http://www.psort.org/psortb/) were used to predict cellular localization. The DNA sequence of *AcTra* is available under the GenBank accession number DQ890522.

## Results

### Comparison of the *AcTra* system with other type IV secretion systems

**Figure 1:**
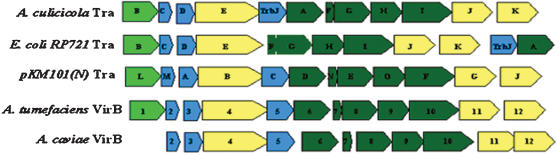
Comparison of AcuTra operon with conjugal and other type IV secretion system homolog. Different colors are indicative of putative role or location of the protein. Light Green – acetyl transglycosylase; Blue – components of pilus assembly; Yellow – NTPases; Dark Green – proteins forming core components.

The sequence analysis showed that *A. culicicola* TFSS operon was 11,061 bp in length, with average G+C content of 47.26% and code for 12 ORFs. We named these ORFs as *TraB, TraC, TraD, TraE, TrbJ, TraA, TraF, TraG, TraH, TraI, TraJ, and TraK* respectively. G+C content of *TraC* is exceptionally high and it is 58%. The *AcTra* gene cluster contains 12 open reading frames (ORFs), homologous to the conjugal transfer system of IncI2 plasmid R721 (Table 1). The arrangement of the genes in *A. culicicola* is different in some respect from those observed in *E. coli*. In case of *A. culicicola*, all 12 proteins were present in continuous stretch whereas in *E. coli*, *TraK* through *TraB* are in sequential manner followed by ~4.5 kb loci coding for some other proteins not associated with TFSS and again followed by *TrbJ* and *TraA*. Arrangement of TFSS genes in *A. culicicola* is very compact with seven overlapping genes. Maximum intergene distance was 39 bases between gene *TraC* and *TraD*. Gene arrangement of *A. culicicola* TFSS and its comparison with other homolog is shown in Fig. 1 whereas the protein analysis details are given in Table 1. The *AcTra* system resembles other type IV secretion systems including the Tra system of plasmids RP721 and pKM101 of *E. coli*, VirB-D4 systems of *A. tumefaciens*, *B. henselae*, *A. caviae* and the Ptl system of *B. pertussis*.

**Table 1:**
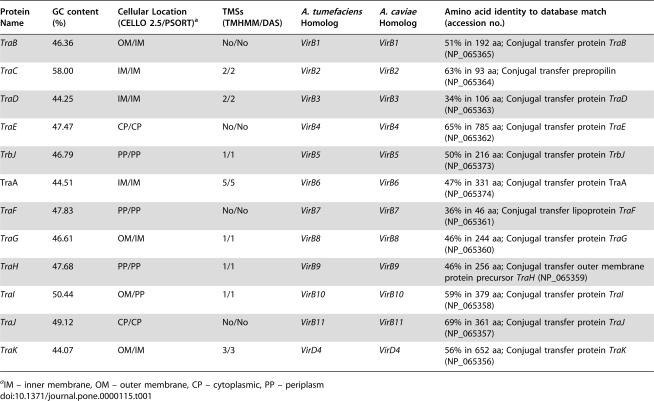
Properties of *A. culicicola* AcTra operon ORFs and their deduced products.

Protein Name	GC content (%)	Cellular Location (CELLO 2.5/PSORT)^a^	TMSs (TMHMM/DAS)	*A. tumefaciens* Homolog	*A. caviae* Homolog	Amino acid identity to database match (accession no.)
*TraB*	46.36	OM/IM	No/No	*VirB1*	*VirB1*	51% in 192 aa; Conjugal transfer protein *TraB* (NP_065365)
*TraC*	58.00	IM/IM	2/2	*VirB2*	*VirB2*	63% in 93 aa; Conjugal transfer prepropilin (NP_065364)
*TraD*	44.25	IM/IM	2/2	*VirB3*	*VirB3*	34% in 106 aa; Conjugal transfer protein *TraD* (NP_065363)
*TraE*	47.47	CP/CP	No/No	*VirB4*	*VirB4*	65% in 785 aa; Conjugal transfer protein *TraE* (NP_065362)
*TrbJ*	46.79	PP/PP	1/1	*VirB5*	*VirB5*	50% in 216 aa: Conjugal transfer protein *TrbJ* (NP_065373)
TraA	44.51	IM/IM	5/5	*VirB6*	*VirB6*	47% in 331 aa; Conjugal transfer protein TraA (NP_065374)
*TraF*	47.83	PP/PP	No/No	*VirB7*	*VirB7*	36% in 46 aa; Conjugal transfer lipoprotein *TraF* (NP_065361)
*TraG*	46.61	OM/IM	1/1	*VirB8*	*VirB8*	46% in 244 aa; Conjugal transfer protein *TraG* (NP_065360)
*TraH*	47.68	PP/PP	1/1	*VirB9*	*VirB9*	46% in 256 aa; Conjugal transfer outer membrane protein precursor *TraH* (NP_065359)
*TraI*	50.44	OM/PP	1/1	*VirB10*	*VirB10*	59% in 379 aa; Conjugal transfer protein *TraI* (NP_065358)
*TraJ*	49.12	CP/CP	No/No	*VirB11*	*VirB11*	69% in 361 aa; Conjugal transfer protein *TraJ* (NP_065357)
*TraK*	44.07	OM/IM	3/3	*VirD4*	*VirD4*	56% in 652 aa; Conjugal transfer protein *TraK* (NP_065356)

a IM – inner membrane, OM – outer membrane, CP – cytoplasmic, PP – periplasm

### Gene Assembly in *A. culicicola*

The proteins of the type IV secretion machinery can be grouped according to their function and/or their cellular location [5], [9].

#### Pilus Assembly Components (*TraC*, *TraD* and *TrbJ*)

The *TraC* is considered as major component of the pilus [14], [24] whereas *TrbJ* protein is known as a minor component of the pilus structure [25] and essential for TFSS virulence. We observed fully conserved L (position 109), Y (position 113), and Q (position 123) in *TrbJ* residues might essential for structural and/or functional aspect of *TrbJ*. Periplasmic form of *TrbJ* is required for translocation of a DNA substrate to the cell surface [26]. The *TraD* has not firmly assigned but its cellular localization suggests that it is a minor component of pilus assembly [27]. *TraD* is a short polypeptide and the multiple alignments for *TraD* homolog revealed a well conserved (NS)-R-P-A-(LM)-X_2_-(GN)-(IV)-P motif, which is slightly different from previous report [14]. It also possesses fully conserved D and L at positions 73 and 77 respectively.

#### Proteins forming core components (TraA, *TraF*-*TraI*)

The *TraA* protein is highly hydrophobic with five predicted TMSs and a large central predicted periplasmic loop whose secondary structure is important for DNA substrate translocation [28]. The *TraA* homologs are relatively poorly conserved with no fully conserved residues. *TraF* is a small lipoprotein, has a signal peptide and no predicted TMS. *TraF* interacts with *TraH* and stabilizes several Tra subunits [29], [30]. *TraG* is an inner membrane protein with an N-terminal TMS. The *TraH* subunit is hydrophilic and possesses three functional domains also reported in other species [31]. The N-terminal periplasmic domain of *TraH* is highly conserved which is required for channel activity and pilus biogenesis [31] whereas; C-terminal plays an important role in interaction with *TraF* [30]. The *TraI* is situated in periplasm and possess one TMS. Hydrophobic C-terminal region of *TraI* is conserved and also possesses coiled-coil structure, which is important for interaction of *TraI* with bitopic *TraG* [32] and *TraF*-*TraH* dimer [33]. N-terminal region of *TraI* is hydrophobic and is poorly conserved whereas C-termini exhibits short sequences of alternating hydrophilic and hydrophobic residues forming putative β-sheet structure. Within this C-terminal region are four fully conserved and additional nine well-conserved glycyl residues as reported before [14]. These observations would have some structural and functional implications.

#### NTPases (*TraE*, *TraJ* and *TraK*)

These protein exhibits highest sequence conservation among TFSS components. The *TraE* subunit of *A. culicicola* does not have any predicted TMS but possesses Walker A and Walker B nucleotide binding domains (P-loop) and is localized in cytoplasm. Sequence analysis of *TraJ* protein reveals the presence of highly conserved hydrophobic domains and typical nucleotide binding domains important for ATPase activity. ATPase activity lies in the hexameric form of *TraJ*, which is stimulated by lipids [34]. The *TraK* also possesses conserved motifs and nucleotide binding domains. *TraK* is situated in the inner membrane and there are three N-terminal TMSs. The ATPase activity of *TraE*, *TraJ* and *TraK* energize TFSS function [9], [24].

The *TraB* is dispensable for TFSS function [35], [36] and not required for transfer of TFSS substrates. *TraB* protein analysis predicted conserved transglycosylase domain [36], [37]. It is basically situated in inner membrane or sometimes on outer membrane also and contributes to channel assembly. Three conserved motifs- a) (VI)-X_7_-(VIL)-E-S, b) (LIF)-X_2_-C-X-(SN)-(LI) and c) S-X-Y, have been observed, previous one at N-terminal region and latter two at relatively central portion of the protein.

### Phylogenetic Relationship

**Figure 2:**
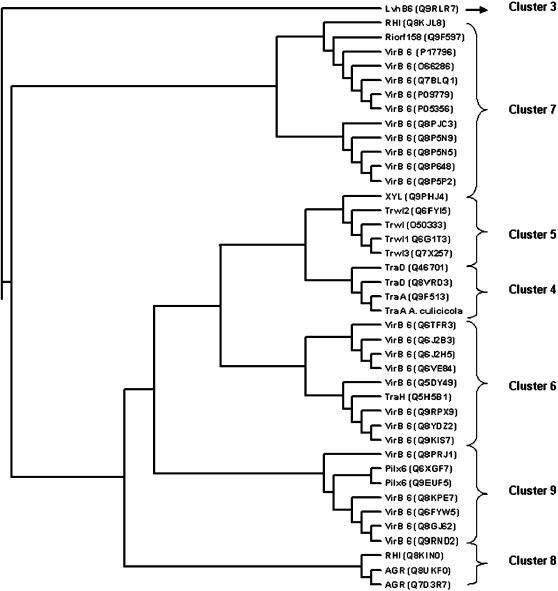
Phylogenetic tree for the *TraA*, drawn using parsimony method of Phylip. Text in parenthesis indicates Accession number of respective gene.

Phylogenetic analysis suggests that the TFSSs have evolved from a common ancestral system with virtually no shuffling of constituents even between sequence-divergent systems. We carried out phylogenetic analysis of A. culicicola TFSS proteins with homologs identified by PSI-BLAST. Trees were generated using phylip with hundred iterations. Topology of all the trees (data not shown) is more or less same and it has been observed that *A. culicicola* grouped with *E. coli* and *Haemophilus influenzae* in cluster 4 proposed by Cao and Saier (Fig. 2) [14].

## Discussion

*A. culicicola* harbors three uncharacterized plasmids. Their role and importance in *A. culicicola* has not determined yet. We investigated conjugal transfer system in one among those plasmids namely pAc3249A. This is the first report of complete analysis of conjugal type IV secretion system in *A. culicicola*. It would be interesting to find the genes present on this plasmid as it has conjugation machinery for self transmission. Our system has shown highest homology to *E. coli* rather than *A. caviae* which indicated that *A. culicicola* might have acquired this plasmid through lateral or horizontal transfer from *E. coli* in mosquito mid-gut, its site of isolation. Complete sequencing and characterization of pAc3249A would reveal the role of conjugal transfer system and eventually the role of pAc3249A in *A. culicicola*.
